# The Serum Treatment in Purpura Hemorrhagica1Read before the Fifteenth Annual Meeting of the Iowa State Veterinary Medical Association, Cedar Rapids, Iowa, January 14 and 15, 1903.

**Published:** 1903-03

**Authors:** J. H. McLeod

**Affiliations:** Charles City, Iowa


### THE SERUM TREATMENT IN PURPURA HEMORRHAGICA.[1]

By J. H. McLeod, D.V.S.,
CHARLES CITY, IOWA.

I cannot recommend as yet the serum treatment in purpura hemorrhagica; however, I can inform you just how far I have gone and the results I have had with this serum.

Case I. Bay horse, driver. The disease developed in a mild form and was doing very well under my usual treatment of stimulants and tonics, but after the fifth day, with the owner's urging, I used the antistreptococcus serum in dose of 30 c.c. The horse was a small driver and I thought this dose enough to start on. It was not necessary to repeat. The horse got along well and made a splendid recovery. No microscopic examination was made in this case, as the symptoms were not grave enough to warrant anything of the kind.

Case II. Almost similar to the former—a well-developed case of purpura—which yielded readily to the serum in doses of 40 c.c., repeated twice.

Case III. Called to treat November 17, 1902, a large draft gelding. Temperature 105°, pulse 90, and respiration about 20. On being moved was stiff all over, and did not care to move unless disturbed, but stood quite still or shifted uneasily on his legs; very large swelling on back and medium sized to small on the breast,

[1] Read before the Fifteenth Annual Meeting of the Iowa State Veterinary Medical Association, Cedar Rapids, Iowa, January 14 and 15, 1903.

abdomen, and groin; mucous membranes blanched with occasional patches of ecchymosis. I promptly informed the owners that the horse would most likely die, but commenced to treat the patient immediately with stimulants and tonics, and advised the use of the antistreptococcus serum, if the price was no object. Injected 40 c.c. the following day at noon, although in the meantime the symptoms had improved since the previous day. The stiffness had lessened considerably, mucous membranes highly injected, and the hemorrhagic spots more typical and plainly visible. The following day: Temperature is normal, pulse is 60 and stronger; the swellings have almost disappeared; the animal is bright and the prospect good for recovery. Next day the animal was improving, and, contrary to my advice, was allowed his liberty, and he even played around outdoors. Friday morning the animal was worse again; stiff in hindquarters, more swelling in groin, with straddling gait; groaned, and seemed in considerable pain at times; patches on mucous membranes gone. The symptoms got gradually worse as the day wore on. Later he was removed into a box-stall, where he cast himself. When relieved, on attempting to rise could not do so. One hour later he got on his feet, but his hind legs refused to support his weight. He had now all the symptoms of azoturia. I passed the catheter and found the urine nearly normal in color, but of higher specific gravity. The prognosis was now very unfavorable. Next morning I could plainly see that the end was near. The attendant informed me that he had passed a large quantity of bloody urine, but he did not save any for my inspection.

As far as the serum treatment in this case is concerned I consider it eminently successful; in fact, more so than in the previous cases, as it was of a much graver nature.

### Results of Four Operations for Cribbing.

Case I. Chestnut gelding, trotter. The operation consisted of section of the sternomaxillaris below where it receives the spinal accessory; also, resection of the sternohyothyroid muscles. This animal made a few unsuccessful attempts after the operation, but gave it up for a time.

Case II. Brown draft stallion. Operation: neurectomy of the spinal accessory; unsuccessful. The owner, however, informs me that he only cribs about half as much, and that his general condition has improved very much.

Case III. Black Percheron stallion. Operation: section of the sternohyothyroid and resection of the spinal accessory; partially successful. The animal attempted the act immediately after the operation, but failed. After three days I found him at times with his mouth held fast to a plank, which he could let go with difficulty. Advised his removal into another stall. This horse was worse when being watched, and at that time next day he performed the act successfully. I ordered his removal into a stall where he could not get hold of anything, and he has not been known to crib since.

CASE IV. Operation same as previous case; successful.

